# Mechanically Robust and Depolymerizable Polyesters With Near‐Complete Monomer Recovery for Circular Additive Manufacturing

**DOI:** 10.1002/adma.73986

**Published:** 2026-07-09

**Authors:** Farzad Gholami, Rampi Ramprasad, H. Jerry Qi

**Affiliations:** ^1^ School of Materials Science and Engineering Georgia Institute of Technology Atlanta Georgia USA; ^2^ George W. Woodruff School of Mechanical Engineering Georgia Institute of Technology Atlanta Georgia USA

**Keywords:** δ‐lactone copolymers, additive manufacturing, depolymerization, recycling, ring‐opening polymerization

## Abstract

Designing polymers that combine tunable macromolecular architecture with complete chemical recyclability remains a fundamental challenge in sustainable polymer chemistry. Here, we report a δ‐lactone copolymer platform synthesized via controlled ring‐opening polymerization of δ‐dodecalactone (δ7) and δ‐valerolactone (δ0), enabling regulation of molecular architecture, mechanical response, and depolymerization pathways within a single polyester chemistry suitable for additive manufacturing (AM; or 3D printing). Segmental programming of δ7 and δ0 domains yields a synergistic combination of chain mobility and reversible crystalline reinforcement, producing recyclable, high‐stiffness 3D‐printing precursors with elastic modulus elevated by 2–3 orders of magnitude relative to prior δ‐lactone materials. End‐group functionalization generates methacrylate‐terminated copolymers and acrylate‐terminated macromonomers that undergo efficient photopolymerization without disrupting the depolymerizable backbone. Blending these components produces photocurable formulations with rheology governed by intrinsic polymer design, enabling compatibility with direct ink writing and digital light processing via temperature‐mediated control of segmental crystallinity. This strategy eliminates external monomers or permanent crosslinkers, preserving chemical integrity and recyclability. Increasing molecular weight shifts the system into an entanglement‐dominated thermoplastic regime for fused filament fabrication. Sequential thermal–catalytic unzipping exploits ceiling‐temperature differences to recover δ7 and δ0 monomers with ∼95% efficiency even from crosslinked networks, with repolymerization yielding materials indistinguishable from virgin polymers.

## Graphical abstract

A sustainable δ‐lactone‐based polymer platform enables multimodal additive manufacturing through DLP, DIW, and FFF, combining tunable crystallinity, photocurable networks, mechanical robustness, and functional response. The printed materials can be mechanically reprocessed and chemically depolymerized into recoverable monomers, demonstrating a closed‐loop strategy for recyclable, high‐performance, and multifunctional 3D‐printed materials.

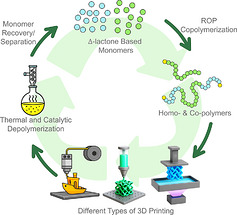

## 1 Introduction

Plastic waste continues to escalate globally, driven not only by the sheer volume of polymers produced but also by the limited effectiveness of recycling methods, which typically degrade material properties and restrict polymers to a short, downcycled life cycle [1, 2, 3]. These limitations have intensified interest in chemistries that enable molecular‐level circularity, in which polymers can be depolymerized and regenerated without loss of performance [4, 5]. This need is particularly pressing in additive manufacturing (AM; or 3D printing), a rapidly expanding technology whose workflow inherently generates substantial waste through failed prints, support structures, and iterative prototyping [6, 7, 8]. Most polymers formulated for AM, whether used in vat photopolymerization, filament extrusion, or ink‐based deposition, are optimized for printability rather than end‐of‐life recovery and are often crosslinked, compositionally complex, or thermally sensitive, rendering them incompatible with mechanical reprocessing or chemical depolymerization [9, 10, 11]. As a result, sustainable 3D printing remains limited to biobased feedstocks or mechanically recycled filaments, approaches that provide only partial mitigation and do not address the fundamental challenge that current 3D printing materials lack, namely, robust, chemically programmable pathways for regeneration. Developing recyclable polymer systems that combine performance, tunability, and true circularity, therefore, remains an unmet need in sustainable manufacturing.

Efforts to develop chemically recyclable materials for AM have led to a diverse set of promising strategies, many of which are tailored to specific printing techniques. In vat photopolymerization or digital light processing (DLP) 3D printing method, recyclable resins based on dynamic covalent polymer networks, including covalent adaptable networks (CANs) [12, 13, 14], as well as other exchangeable or dissociative covalent chemistries [6, 15, 16], or low ceiling‐temperature polyesters [17] have enabled post‐use reprocessing or, in selected cases, recovery of monomer feedstocks. Despite these advances, many dominant recyclable resin strategies in DLP 3D printing exhibit intrinsic limitations that limit their contribution to true chemical circularity. In particular, CANs enable reshaping and reprocessing through dynamic bond exchange but retain a permanent polymer network and do not regenerate monomer feedstocks, restricting circularity to polymer reprocessing rather than monomer‐level recovery [14, 18]. Disulfide‐based photopolymer resins provide an elegant route to dynamic and potentially closed‐loop recycling; however, their practical implementation often requires carefully balanced formulations to suppress premature network formation, and depolymerization can yield residual oligomeric species or require specific catalytic or solvent conditions, complicating robustness and scalability [6]. Low ceiling‐temperature polyesters have emerged as a promising route toward chemically depolymerizable photopolymer resins and have been successfully implemented in DLP 3D printing, as demonstrated by Yue et al. [17]. In these systems, the synthesized materials usually have a low glass transition temperature and exhibit very soft mechanical properties, which do not meet the requirements of applications that require high stiffness. Moreover, because these polyesters alone do not form the permanently crosslinked networks required for resin‐based photopolymerization, printable formulations incorporate additional photocurable components or reactive diluents, typically requiring at least ∼15 wt.% acrylate based monomers or crosslinkers to achieve sufficient network formation. Because these auxiliary components do not undergo depolymerization, monomer recovery is limited to the low‐ceiling‐temperature polyester fraction, and regeneration of the original resin formulation requires the addition of fresh materials. Recent advances in direct ink writing (DIW) 3D printing have produced reprocessable or recyclable inks and composites, most commonly based on CANs [19, 20, 21]. These systems leverage reversible bond exchange to enable reshaping and reprocessing after printing while retaining a permanently crosslinked network. However, similar to DLP 3D printing, such approaches primarily support polymer‐level reprocessing rather than depolymerization to recover monomeric feedstocks, thereby limiting chemical circularity.

In fused filament fabrication (FFF) 3D printing, widely adopted materials such as polylactic acid, derived from renewable feedstocks, and mechanically recycled commodity polymers sourced from post‐consumer waste [22, 23, 24], but their circularity is primarily achieved through thermal reprocessing, which can lead to gradual property degradation and does not enable monomer‐level chemical recovery [25]. More broadly, the realization of practical circular manufacturing benefits from materials that not only depolymerize efficiently but also retain mechanical integrity and processability across multiple recycling cycles. Across various 3D printing methods, many depolymerizable or recyclable polymer systems favor chemical reversibility or processability at the expense of stiffness, toughness, or melt strength, which can limit their applicability in load‐bearing or structurally demanding components. Recent work by Su et al. [26]. demonstrates an important advance in this direction by developing a chemically recyclable thermoplastic via reversible Thia‐Michael reaction specifically tailored for filament‐based printing, combining strong mechanical performance with excellent melt processability. However, this system relies on cyclic thioenone monomers prepared through multistep organic synthesis involving specialized reagents and subsequent functionalization, which introduces additional complexity relative to more conventional polymer feedstocks [26]. This highlights an ongoing challenge for chemically recyclable filament materials: achieving high‐performance, reprocessable polymers while maintaining synthetically efficient and scalable monomer production suitable for widespread manufacturing.

**FIGURE 1:**
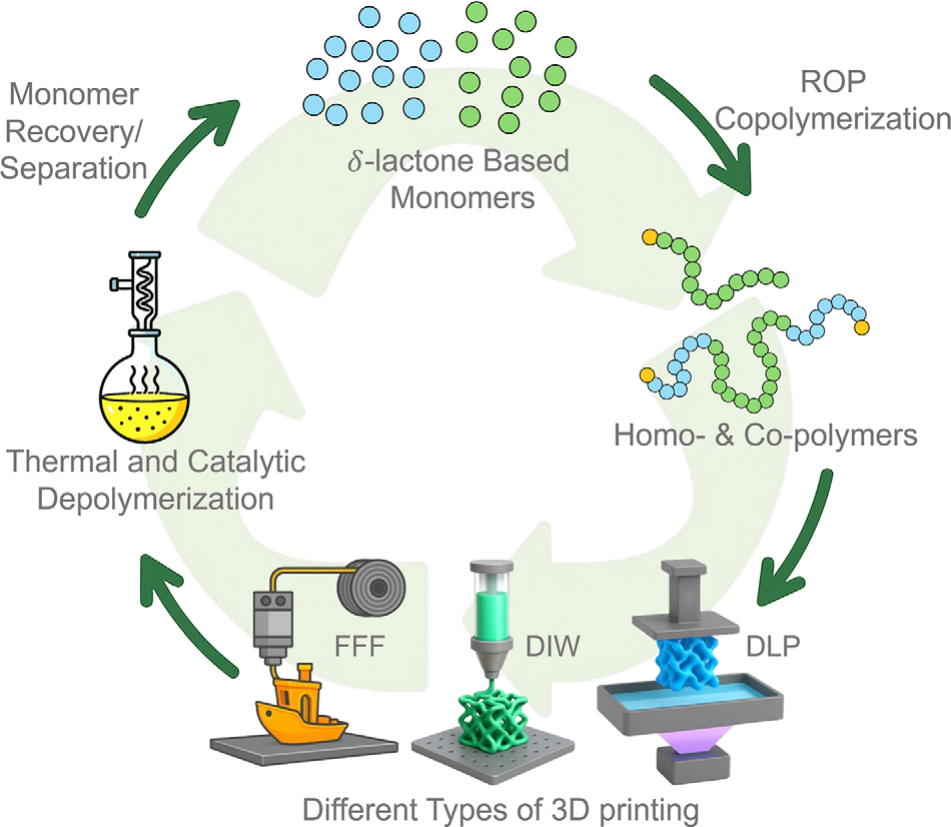
Circular workflow for δ‐lactone based polymer systems enabling direct ink writing (DIW), digital light processing (DLP), and filament fused fabrication (FFF) 3D printing and subsequent monomer recovery.

To address the aforementioned challenges, we develop a depolymerizable δ‐lactone copolymer platform that enables systematic control over molecular architecture and phase behavior while preserving chemical circularity. Ring opening polymerization (ROP) of δ‐dodecalactone (δ7) and δ‐valerolactone (δ0) produces block copolymers whose composition and molecular weight can be tuned to adjust amorphous and crystalline domain formation, chain entanglement, and mechanical strength, providing access to a broad range of thermomechanical responses within a single backbone chemistry. On one hand, δ7 has low ceiling‐temperature, thereby making the resulting polyesters thermodynamically favored to depolymerize at elevated temperatures yet stable under service conditions [27, 28]. On the other hand, δ0, due to the lack of pendant chains, demonstrates a high degree of crystallinity, improving the mechanical properties [29, 30, 31]. Hydroxyl end groups permit selective methacrylation to generate photocurable formulations, and acrylate‐initiated polymerization of δ7 with relatively low molecular weight (MW) produces low viscosity macromonomers which serve as fully reactive diluents. By blending these components, the same chemically recyclable platform can be formulated as photoreactive resin and viscoelastic inks for DLP and DIW 3D printing, respectively. In addition, increasing the copolymer MW enhances physical chain entanglement, thereby increasing melt strength to a level sufficient for extrusion [32, 33, 34], enabling filament fabrication for FFF 3D printing. These demonstrate that the depolymerizable δ‐lactone platform can be strategically programmed to meet the distinct processing and performance requirements of multiple AM techniques without changing the underlying polymer chemistry. Furthermore, this tunability enables the fabrication of materials with tailored mechanical properties alongside functional responses, including electrically conductive and sensing‐capable architectures, across multiple additive manufacturing modalities within a single chemically recyclable platform. Importantly, due to the nature of the polymer backbones, the copolymers can undergo a sequential thermal–catalytic depolymerization procedure in which δ7 is recovered in a first, purely thermal step, followed by catalytic depolymerization of δ0, producing separately collected monomers with high purity, suitable for repolymerization. These recovered monomers can be directly repolymerized to regenerate copolymers with comparable molecular weights and thermomechanical properties, demonstrating preservation of material quality across recycling cycles. This integrated workflow, summarized in Figure 1, establishes a continuous route from monomer synthesis to various AM techniques and back to monomer recovery and separation.

## 2 Results

### 2.1 Homo‐ and Co‐Polymer Synthesis

**FIGURE 2:**
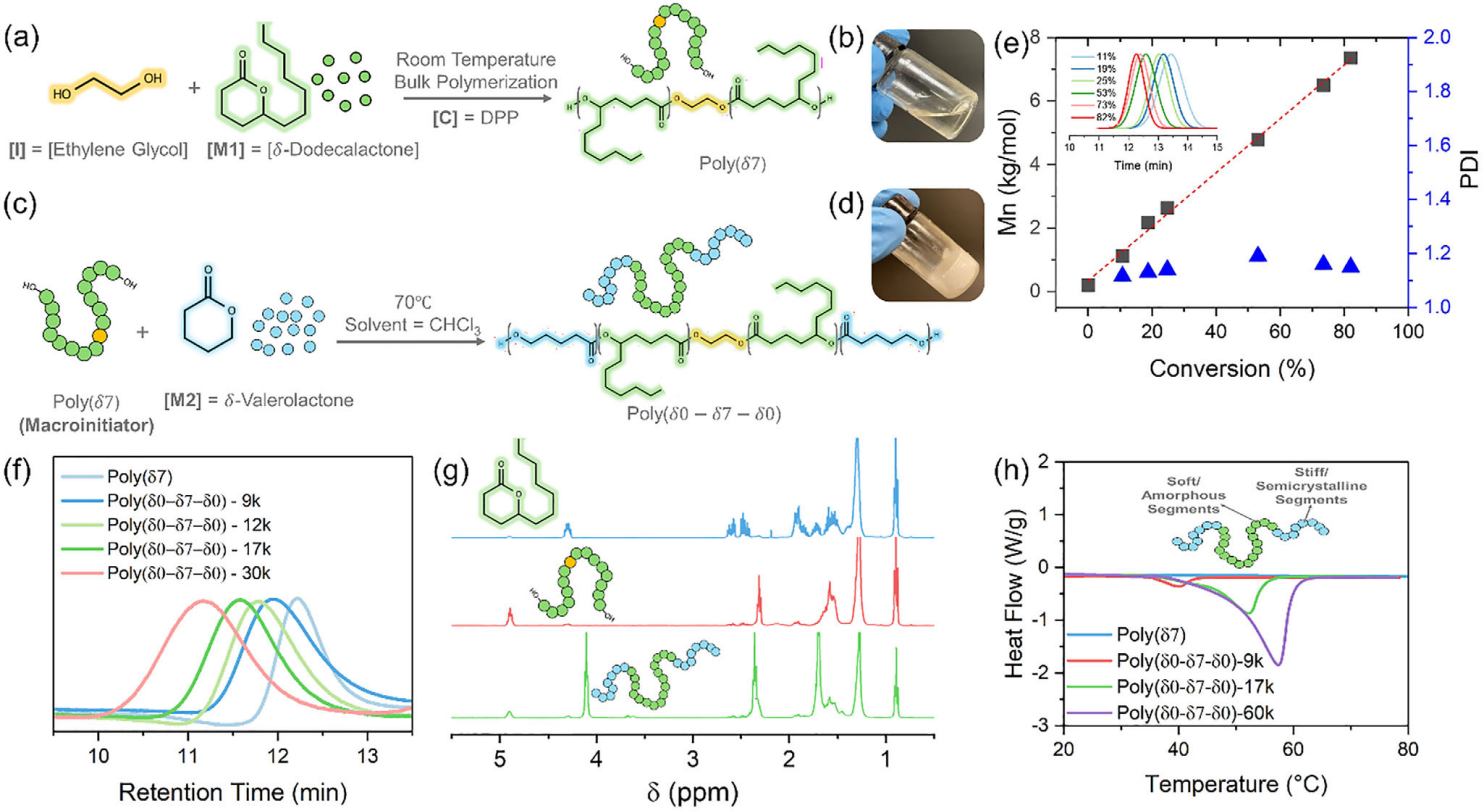
Synthesis details of homopolymers and copolymers. (a) ROP synthesis route of poly(δ7) homopolymer of EG diol; (b) synthesized homopolymer as an amorphous, viscose, and transparent liquid. (c) ROP synthesis route of poly(δ0‐δ7‐δ0) copolymer on poly(δ7) as macroinitiator; (d) synthesized copolymer as a semi‐crystalline and translucent solid. (e) Mn and Đ vs. conversion of ROP of poly(δ7) (GPC curves for different samples are presented at index figure). (f) Retention time graphs obtained from GPC measurement for poly(δ7) and poly(δ0‐δ7‐δ0) copolymers with different targeted molecular weights. (g) ^1^H‐NMR spectra for monomer (δ7), homopolymer (poly(δ7)), and copolymer (poly(δ0‐δ7‐δ0)). (h) DSC thermograms of poly(δ7) and poly(δ0‐δ7‐δ0) copolymers with different molecular weight (Exo‐up graph).

To establish a depolymerizable polyester backbone with controlled architecture, we first synthesize poly(δ7) homopolymers via bulk ring‐opening polymerization (ROP) from hydroxyl‐functional initiators (Figure 2a). Because lactone ROP is highly sensitive to water, all reactions are performed under anhydrous nitrogen conditions to prevent competitive initiation and preserve molecular‐weight control. Polymerizations are catalyzed by diphenyl phosphate (DPP), an organocatalyst that enables efficient δ‐lactone ROP at room temperature [35], allowing precise tuning of the targeted degree of polymerization by varying the monomer‐to‐initiator ratio (Table 1). Two initiator types are employed to access complementary precursor structures. As illustrated in Figure 2a, ethylene glycol (EG) served as a difunctional initiator, yielding telechelic α,ω‐dihydroxyl poly(δ7) chains suitable for subsequent chain‐extension and block‐copolymer formation. In parallel, 2‐hydroxyethyl acrylate (2‐HEA) is used as a monofunctional initiator, producing poly(δ7) macromonomers in which the terminal acrylate remains available for downstream photocuring. The proton nuclear magnetic resonance Spectroscopy (^1^H NMR) spectrum of Poly(δ7)‐2HEA is presented in Figure S1, confirming the successful synthesis of this polymer. Analysis of the ^1^H NMR spectrum reveals distinct signals corresponding to the acrylate functional group in the 5.5–6.5 ppm range, alongside the characteristic resonances associated with poly(δ7). The resulting poly(δ7) homopolymer formed a clear, viscous polyester, consistent with the behavior of an amorphous δ‐lactone polymer (Figure 2b).

**TABLE 1:** Synthesis details and conditions of different homopolymers and copolymers with different molecular weight and architecture.

Sample	Initiator	Catalyst	Monomer	[M]:[I]:[C](Mole ratio)	Solvent	Temperature(°C)	Mn(kg.mol^−1^)	Ð
Poly(δ7)‐ EG – 7k	EG	DPP	δ7	40:1:0.4	−	R.T.	7.2 ± 0.2	1.18 ± 0.01
Poly(δ7)‐ EG – 20k	EG	DPP	δ7	120:1:1.2	−	R.T.	19.7 ± 0.7	1.16 ± 0.01
Poly(δ7)‐ 2HEA – 2k	2‐HEA	DPP	δ7	12:1:0.12	−	R.T.	1.9 ± 0.2	1.25 ± 0.03
Poly(δ7)‐ 2HEA – 4k	2‐HEA	DPP	δ7	25:1:0.12	−	R.T.	4.2 ± 0.5	1.28 ± 0.01
Poly(δ0‐δ7‐δ0) – 9k	Poly(δ7)‐ EG – 7k	−	δ0	25:1:0	CHCl_3_	70	9.1 ± 1	1.14 ± 0
Poly(δ0‐δ7‐δ0) – 12k	Poly(δ7)‐ EG – 7k	−	δ0	60:1:0	CHCl_3_	70	12.3 ± 1.1	1.21 ± 0.02
Poly(δ0‐δ7‐δ0) – 17k	Poly(δ7)‐ EG – 7k	−	δ0	100:1:0	CHCl_3_	70	17.5 ± 0.5	1.18 ± 0.01
Poly(δ0‐δ7‐δ0) – 30k	Poly(δ7)‐ EG – 7k	−	δ0	250:1:0	CHCl_3_	70	31 ± 2.1	1.2 ± 0.04
Poly(δ0‐δ7‐δ0) – 60k	Poly(δ7)‐ EG – 20k	−	δ0	450:1:0	CHCl_3_	70	59.8 ± 3.5	1.19 ± 0.02

To introduce semicrystalline domains into the polymer architecture, the telechelic poly(δ7) is employed as a macroinitiator for δ0 ring‐opening polymerization (Figure 2c). In contrast to the long‐alkyl δ7 repeat units, which yield fully amorphous chains, δ0 contains no pendant substituents and therefore forms highly regular sequences capable of crystallization [29, 36]. This difference in stereoregularity enables δ0 incorporation to serve as a hard‐segment motif within the polyester framework. Similar to homopolymerization, the copolymerization process is conducted under dry nitrogen. Chain extension proceeds directly from the hydroxyl termini of poly(δ7) without the need for additional initiators, enabling clean growth of the δ0 segment while preserving end‐group fidelity. Unlike the room‐temperature bulk polymerization of δ7, δ0 incorporation rapidly increases mixture viscosity due to the emerging semicrystalline character [34]. To maintain homogeneity during chain growth, δ0 polymerization is carried out in chloroform solvent at 70°C, which ensures adequate solvation and uniform block extension. Under these conditions, growth of the δ0 block reproducibly produces a translucent white solid (Figure 2d), consistent with formation of semicrystalline δ0‐containing copolymers. The resulting poly(δ0‐δ7‐δ0) materials thus integrate an amorphous midblock with crystallizable end segments, providing a tunable hard–soft architecture central to the mechanical performance of the platform. Table 1 summarizes the reaction conditions and the molar ratios of reagents for the different homo‐ and copolymer systems.

The polymerization kinetics of δ7 are quantified by pairing real‐time conversion measurements with molecular‐weight evolution, as shown in Figure 2e. At defined intervals, small aliquots of the reaction mixture are withdrawn and analyzed directly, without quenching or purification, so that each sample accurately reflects the chemical state at that moment. Monomer conversion is determined from ^1^H‐NMR by tracking the decrease in the characteristic δ7 resonances (2.4 – 2.7 ppm), and the same aliquots are evaluated by gel permeation chromatography (GPC) to measure the accompanying changes in molecular weight. The complete set of ^1^H NMR spectra along with peak integrals for these time points is provided in Figures S2–S8. The resulting M_n_ vs. conversion relationship exhibits an almost linear trend, indicating uniform initiation together with a well‐controlled chain‐growth process. Dispersity (Đ) remains low throughout the reaction (<1.2), indicating that transesterification, chain transfer, and other side reactions are effectively suppressed under the applied organocatalytic conditions. The corresponding GPC traces show a smooth, unimodal progression toward shorter retention times, with no shoulders or secondary features, confirming synchronized growth across the polymer chain population. These dispersity combined ^1^H‐NMR and GPC measurements establish that δ7 undergoes controlled ROP under the selected conditions and produces a family of poly(δ7) homopolymers whose molecular weight can be adjusted predictably for use as macroinitiators in subsequent copolymerization steps. For clarity, a stacked representation of the time‐evolution ^1^H NMR spectra, showing the progressive disappearance of δ7 monomer peaks and emergence of poly(δ7) polymer resonances, is presented Figure S16a.

Successful chain extension during δ0 copolymerization is evidenced by the systematic evolution of the molecular‐weight distributions shown in Figure 2f. GPC of the resulting poly(δ0‐δ7‐δ0) copolymers exhibits unimodal traces that shift progressively to lower retention times with increasing targeted molecular weight, consistent with uniform growth from the poly(δ7) macroinitiator. The absence of residual macroinitiator peaks or high‐molecular‐weight shoulders indicates efficient incorporation of δ0 segments and minimal side reactions during block formation. This monotonic molar‐mass evolution demonstrates that copolymer molecular weight can be predictably tuned by varying the δ0 feed composition while preserving narrow distributions (Đ < 1.3). Such control enables systematic modulation of chain architecture and, consequently, access to materials with distinct thermal and mechanical responses within a single depolymerizable polyester platform. Consistent with the molar‐mass evolution observed by GPC, δ0 incorporation during chain extension is independently confirmed by ^1^H NMR. The individual time‐resolved spectra and corresponding peak integrations used to quantify δ0 conversion (based on monitoring the characteristic δ0 monomer resonance at 1.8–2.0 ppm) are provided in Figures S9–S15, while a stacked representation illustrating the progressive disappearance of monomer resonances and emergence of polymer signals is shown in Figure S16b. The sequential nature of the homopolymerization and subsequent chain‐extension process is further illustrated by time‐resolved conversion profiles derived from ^1^H‐NMR (Figure S17). δ7 polymerization proceeds steadily to approximately 85% conversion over 36 h, after which δ0 is introduced and undergoes rapid incorporation, reaching near‐quantitative conversion within an additional 36 h.

The successful formation of both the poly(δ7) homopolymer and the δ0‐extended copolymer is further confirmed by ^1^H NMR spectroscopy (Figure 2g). The spectrum of poly(δ7) shows the disappearance of the δ7 monomer resonances (2.4 – 2.7 ppm) accompanied by the emergence of broadened polyester signals characteristic of the polymer backbone, consistent with efficient ROP. Following chain extension, additional resonances corresponding to the δ0 repeat units appear in the copolymer spectrum, while the original poly(δ7) signals are retained, confirming incorporation of δ0 segments rather than formation of a separate homopolymer population. The absence of residual monomer peaks and the simultaneous presence of both δ7 and δ0 backbone signals verify successful sequential polymerization and formation of the targeted poly(δ0‐δ7‐δ0) architecture. The ^1^H NMR spectra for δ7 monomer, poly(δ7) homopolymer, δ0 monomer, and poly(δ0‐δ7‐δ0) copolymer with details and assigned peaks are presented in Figure S18a–d, respectively. The incorporation of δ0 segments and the resulting evolution of polymer architecture are reflected in the thermal behavior of the materials, as shown by differential scanning calorimetry (DSC) in Figure 2h. The poly(δ7) homopolymer exhibits no detectable thermal transitions over the measured temperature range, consistent with its fully amorphous character. In contrast, the poly(δ0–δ7–δ0) copolymers display well‐defined melting endotherms whose magnitude and position depend on molecular weight, indicating the formation of semicrystalline δ0 domains within the polyester backbone. As copolymer molecular weight increases, the melting transition becomes more pronounced (T_m_ of increases from ∼40°C for poly poly(δ0–δ7–δ0)‐9k to ∼60°C for poly(δ0–δ7–δ0)‐60k), reflecting enhanced crystallizable segment length and improved organization of the δ0 domains. These results confirm that sequential δ0 incorporation introduces thermally distinct hard segments into an otherwise amorphous poly(δ7) matrix, establishing a tunable soft–hard architecture. Mechanical and physical properties of the copolymers can be tuned by changing the ratio of hard and soft segments during synthesis. In addition to the emergence of semicrystalline melting transitions, changes in the amorphous‐phase dynamics are evaluated by DSC. As shown in Figure S19, the glass transition temperature (T_g_) systematically shifts to higher temperatures with increasing copolymer molecular weight. The poly(δ7) homopolymer exhibits a T_g_ near −72°C, consistent with its highly flexible, long‐alkyl polyester backbone [37]. Incorporation of δ0 segments and progressive chain extension result in a gradual increase in T_g_, reaching approximately −60°C for the highest‐molecular‐weight poly(δ0–δ7–δ0)‐60k copolymer. This upward shift reflects increased restriction of segmental mobility in the amorphous regions, arising from both higher molecular weight and the physical confinement imposed by semicrystalline δ0 domains [38]. Notably, despite the presence of hard segments, the T_g_ remains well below ambient temperature across all compositions, preserving elastomeric behavior while enabling tunable mechanical reinforcement through crystalline‐domain formation [39].

### 2.2 Photocurable Functionalization

**FIGURE 3:**
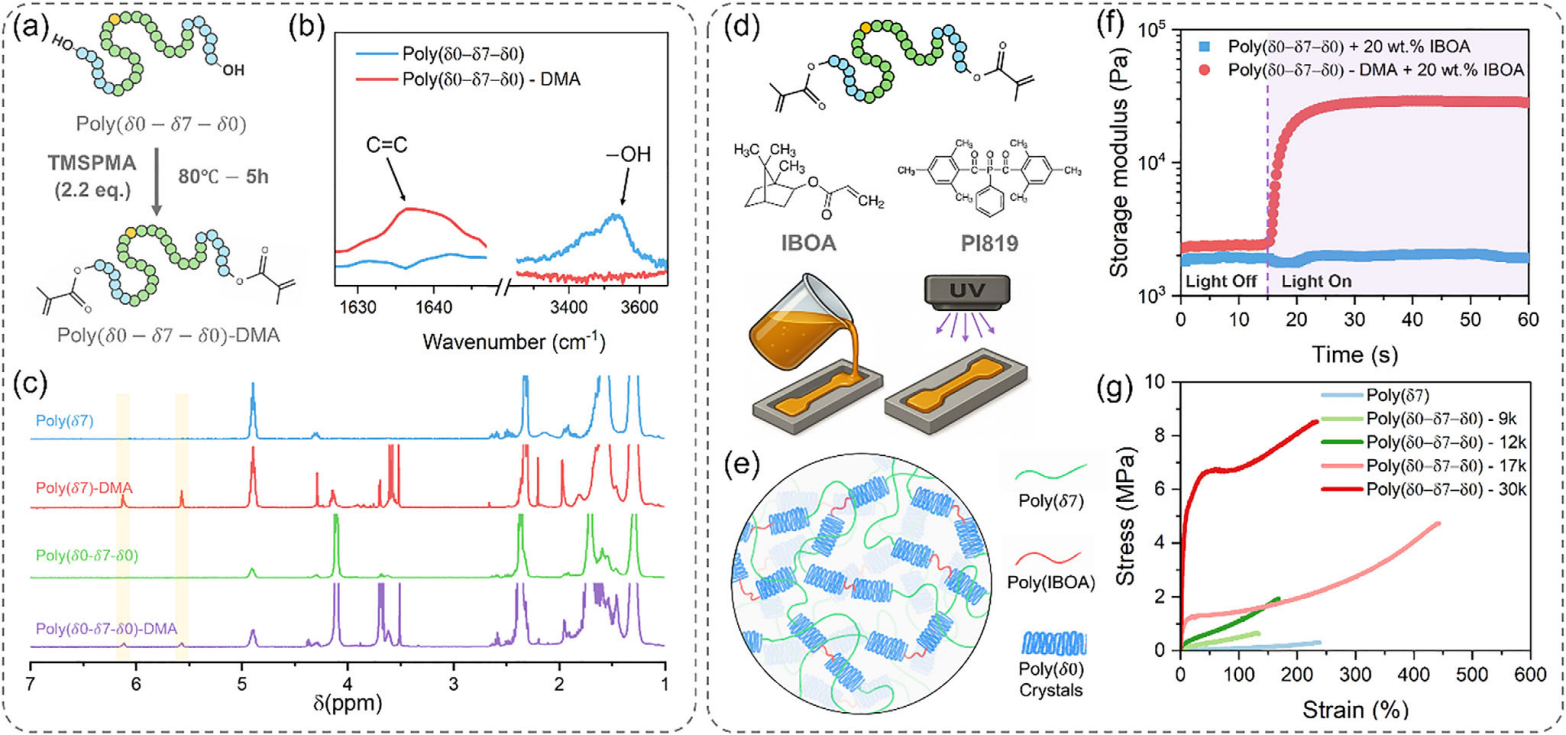
Functionalization of homopolymer and copolymers for fabrication of photo‐curable polymers for light‐based 3D printing techniques. (a) Dimethacrylate functionalization of lactone‐based homo‐ and copolymers using TMSPMA through a condensation reaction. (b) FTIR spectra of the poly(δ0–δ7–δ0) copolymer before and after dimethacrylation, highlighting changes in the carbonyl and hydroxyl regions. (c) ^1^H‐NMR spectra of the poly(δ0–δ7–δ0) copolymer before and after dimethacrylation, showing the appearance of methacrylate characteristic peaks after modification. (d) Incorporation of poly(δ0–δ7–δ0)‐DMA as a multifunctional crosslinker within photocurable resin formulations. (e) Schematic illustrating crystalline δ0 segments embedded within the crosslinked network that contribute to enhanced mechanical performance. (f) Photorheology of poly(δ0–δ7–δ0) before and after dimethacrylation and in the presence of 20 wt.% IBOA, showing light‐induced gelation behavior. (g) Tensile stress–strain curves of cured poly(δ7) and poly(δ0–δ7–δ0) networks with different molecular weights formulated with 20 wt.% IBOA.

To introduce photocurable functionality while preserving the depolymerizable polyester backbone, the terminal hydroxyl groups of telechelic poly(δ7) homopolymers and poly(δ0–δ7–δ0) copolymers (polymerized on EG) are functionalized with methacrylate end groups using 3‐(trimethoxysilyl)propyl methacrylate (TMSPMA) [40, 41, 42]. This end‐group modification installs photopolymerizable methacrylate functionalities at the chain termini without altering the δ‐lactone repeat units, thereby retaining the intrinsic depolymerization capability of the backbone while enabling subsequent photochemical crosslinking. The reaction is carried out under mild thermal conditions (80°C) for 5 h, consistent with preferential activation of terminal hydroxyl groups rather than transesterification or backbone modification (Figure 3a). The success of the end‐group methacrylation is evaluated by FTIR spectroscopy (Figure 3b). The parent poly(δ0–δ7–δ0) copolymer exhibits a broad absorption centered at ∼3400 cm^−^^1^, attributed to the stretching vibration of terminal hydroxyl groups [43]. Upon functionalization with TMSPMA, this hydroxyl‐associated band is markedly attenuated, accompanied by the appearance of a new absorption at ∼1640 cm^−^^1^ corresponding to the ester carbonyl of the methacrylate moiety [44]. Further confirmation is provided by ^1^H NMR spectroscopy as shown in Figure 3c. In contrast to the unmodified copolymer, the methacrylated material displays two distinct resonances at approximately 5.5 and 6.1 ppm [45] (highlighted), assigned to the vinyl protons of the methacrylate group. The emergence of these signals provides clear spectroscopic evidence for the successful installation of methacrylate functionalities at the polymer chain ends. Further confirmation of the reaction progress is obtained by time‐dependent ^1^H NMR spectroscopy. To evaluate the progress of the functionalization reaction, aliquots are collected during the TMSPMA reaction and analyzed by ^1^H NMR after precipitation in methanol to remove unreacted TMSPMA reagents. The corresponding ^1^H NMR spectra, including the integrated methacrylate vinyl proton peaks, are presented in Figure S20a–e. The integrated intensity of the methacrylate vinyl proton signals at 6.1 ppm increases with reaction time and reaches a plateau after 5 h, with no significant increase observed between 5 and 10 h. These results indicate that efficient end‐group functionalization is achieved within 5 h. Extending the reaction beyond this period does not result in a measurable increase in functionalization, and prolonged reaction times exceeding 18 h could lead to the onset of network formation due to thermal activation of methacrylate end‐groups.

The methacrylate‐functionalized δ‐lactone polymers, irrespective of molecular architecture, possess two polymerizable methacrylate groups located at the chain termini, enabling their use as macromolecular crosslinkers in photocurable resin formulations. To evaluate this capability, the functionalized polymers are blended with 20 wt.% isobornyl acrylate (IBOA), a monofunctional reactive diluent commonly employed to enhance photopolymerization kinetics and network formation. As schematically shown in Figure 3d, the resulting formulations are cast and subsequently cured under UV irradiation to generate crosslinked polymer networks. Owing to the semicrystalline nature of the poly(δ0–δ7–δ0) copolymer backbone, UV curing induces the formation of microcrystalline domains within the crosslinked network, which act as physical reinforcement points and contribute to the enhanced mechanical performance of the cured materials [46] (Figure 3e). This structural feature distinguishes the functionalized copolymers from conventional amorphous photopolymers and provides an additional mechanism for enhancing mechanical properties beyond covalent crosslinking. The photopolymerization behavior of the methacrylated copolymer is further examined by photorheology and compared against an otherwise identical formulation containing the unmodified poly(δ0‐δ7‐δ0) copolymer as a reference. The storage modulus (G′) vs. time plot is presented in Figure 3f. Upon UV exposure, the methacrylated system exhibits a rapid and pronounced increase in storage modulus, indicative of efficient network formation driven by the terminal methacrylate groups. In contrast, blends containing the unmodified copolymer show only a minor modulus increase. This response can be attributed to IBOA homopolymerization, but at only 20 wt.%, IBOA is insufficient to establish a continuous crosslinked network. This distinct rheological response confirms that the introduced methacrylate functionalities remain readily accessible and highly reactive under UV irradiation, effectively transforming the depolymerizable copolymer into an efficient, UV‐responsive network former. Furthermore, the loss modulus (G′′) and tan δ graphs for these sample are provided in Figure S21a,b, respectively.

The mechanical properties of the cured materials further demonstrate the architectural tunability imparted by the poly(δ0‐δ7‐δ0) backbone. Increasing the molecular weight of the copolymer precursor increases both the length and effective population of semicrystalline δ0 segments, yielding photocured networks with enhanced stiffness, strength, and energy dissipation, as illustrated in Figure 3g (length of δ7 segment is constant and is 7.2 kg/mol for all of these samples). Low–molecular–weight copolymers form soft, highly extensible networks dominated by amorphous chain segments, whereas higher–molecular–weight analogues exhibit significant strain hardening and substantially high strength, due to presence of crystalline microdomains. While the formulation composition is held constant at a fixed polymer‐to‐IBOA weight ratio, variations in copolymer molecular weight alter the effective network architecture by changing the chain length between crosslinks and the extent of segmental crystallinity. As a result, the observed mechanical differences arise primarily from changes in polymer architecture and crystalline reinforcement rather than from differences in formulation chemistry. These results demonstrate that methacrylated poly(δ0‐δ7‐δ0) copolymers function as segment‐tunable, depolymerizable crosslinkers, enabling photocured networks whose elastic modulus can be systematically adjusted over a broad range (from 0.140 to 160 MPa) while maintaining rapid UV responsiveness and chemical recyclability. Table S1 provides details of the mechanical properties of these samples, including Young's modulus and elongation at break.

To evaluate the contribution of the high‐T_g_ IBOA component, control formulations are prepared by replacing IBOA with butyl acrylate (BA), an intrinsically softer acrylate monomer. As illustrated in Figure S22, although the BA‐based networks exhibit lower absolute moduli than the corresponding IBOA‐based materials, the modulus still increases substantially from 90 ± 12 kPa for Poly(δ7) + 20 wt.% BA to 5.3 ± 0.4 MPa for Poly(δ0‐δ7‐δ0)‐30k + 20 wt.% BA. This result confirms that IBOA contributes to network rigidity, while also demonstrating that the crystalline δ0‐containing copolymer architecture provides significant reinforcement independent of the high‐T_g_ IBOA component.

### 2.3 Processing‐Tunable Photocurable Resins for DIW and DLP

**FIGURE 4:**
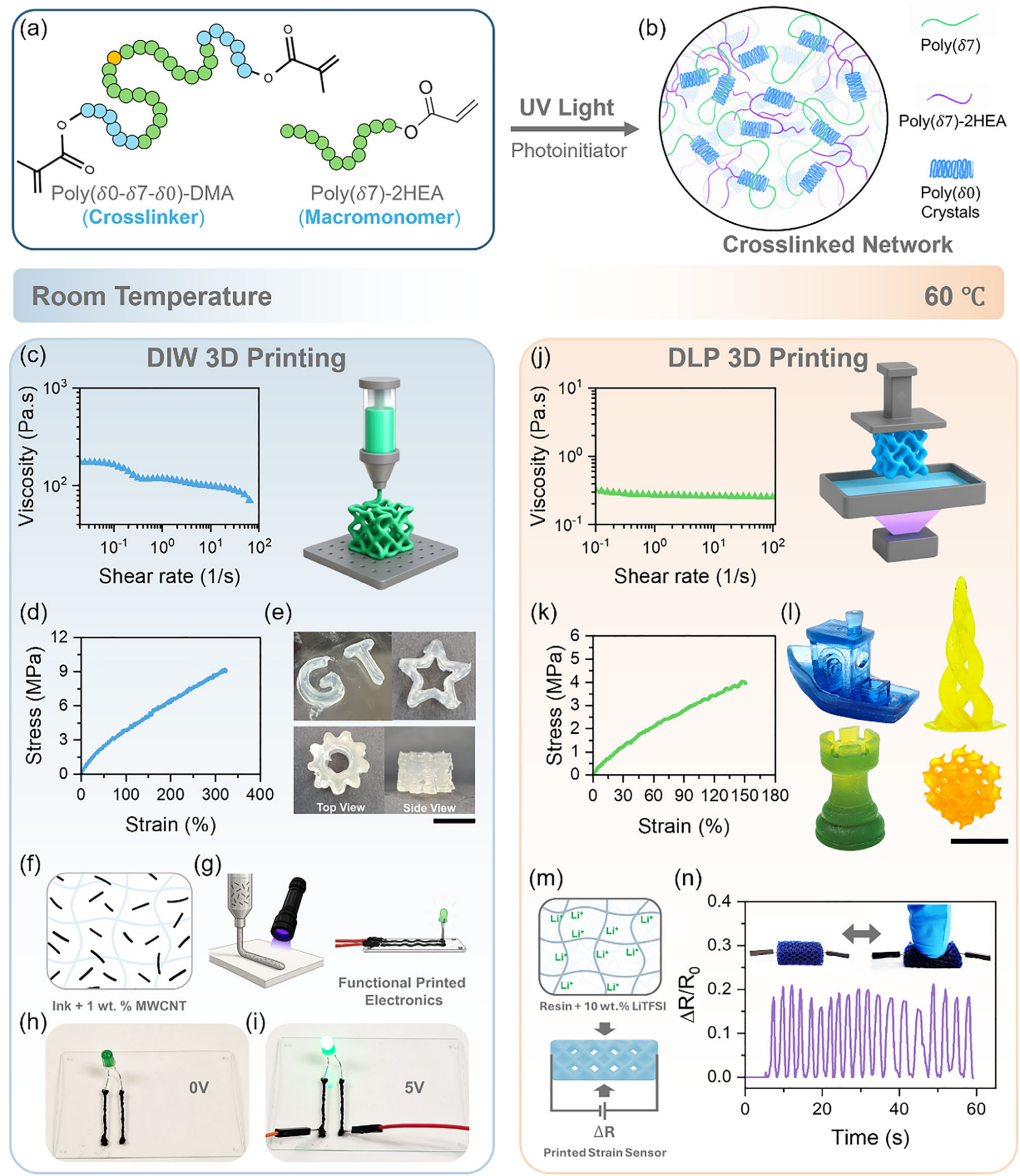
Photocurable and fully depolymerizable material platform for 3D printing. (a) Schematic representation of the main components in the prepared ink: poly(δ0–δ7–δ0)‐DMA as the crosslinker and poly(δ7)‐2HEA as the macromonomer. (b) Formation of a crosslinked network upon photopolymerization under UV light; different polymer chain segments are color‐coded in the schematic. (c) Viscosity as a function of shear rate for the formulation at room temperature, falling within the rheological window suitable for DIW inks. (d) Stress–strain curve of the crosslinked DIW material, demonstrating robust mechanical performance. (e) DIW‐printed 3D objects illustrating ink printability (scale bar: 10 mm). (f) Schematic illustration of the DIW ink containing 1 wt.% MWCNT to impart electrical conductivity, and (g) corresponding printed conductive traces. DIW‐printed conductive traces assembled as a simple circuit (h) before and (i) after connection to a 5 V power supply to illuminate an LED. (j) Viscosity as a function of shear rate for the formulation at 60°C, within the viscosity range required for DLP resins. (k) Stress–strain curve of the crosslinked DLP material, indicating robust mechanical properties. (l) DLP‐printed 3D objects demonstrating resin printability (scale bar: 10 mm). (m) Schematic representation of a DLP resin containing 10 wt.% LiTFSI as an ionic conductor and the resulting printed conductive lattice functioning as a strain sensor. (n) Normalized resistance change (ΔR/R_0_) of the printed strain sensor during cyclic loading–unloading.

Building on the demonstrated ability of methacrylated poly(δ0–δ7–δ0) copolymers to function as segment‐tunable photocurable crosslinkers, we next use this platform to formulate resins compatible with multiple 3D printing techniques. The photocurable functionality of these polymers enables their formulation as inks suitable for DIW and DLP 3D printing. The formulations consist of binary mixtures of poly(δ0–δ7–δ0)‐DMA copolymers and a low–molecular‐weight acrylate‐terminated poly(δ7)‐2HEA macromonomer, as shown in Figure 4a. The poly(δ0–δ7–δ0)‐DMA functions as a macromolecular crosslinker, introducing mechanical integrity and segmental reinforcement through δ0‐rich semicrystalline domains, while the poly(δ7)‐2HEA component serves as a fully reactive diluent that enables viscosity control and homogeneous flow. Importantly, both components share the same depolymerizable δ‐lactone polyester backbone, ensuring that processability is tuned through polymer architecture rather than the incorporation of chemically distinct, non‐depolymerizable additives. Upon UV exposure, acrylate and methacrylate groups undergo radical crosslinking to form a polyester network in which semicrystalline δ0 domains are embedded within an amorphous matrix (Figure 4b). As established in the preceding section, these crystalline microdomains provide physical reinforcement while remaining thermally reversible. The reversible melting of δ0 segments at elevated temperatures introduces temperature‐dependent mobility in the uncured state, enabling modulation of flow behavior without altering formulation composition. By adjusting molecular weight, composition, and processing temperature, this material can be tuned to meet the rheological and curing requirements of both DIW and DLP. The following sections demonstrate how this material design is implemented across these two 3D printing techniques.

At room temperature, the resin containing a high concentration of high‐molecular‐weight poly(δ0‐δ7‐δ0)‐DMA exhibits viscoelastic behavior due to the presence of semicrystalline δ0‐rich segments that remain solid under ambient conditions. As shown in Figure 4c, the uncured poly(δ0‐δ7‐δ0)‐based inks exhibit shear‐thinning viscosity. This behavior enables extrusion at room temperature without thermal assistance or solvent addition while retaining sufficient elastic recovery after deposition to support multilayer DIW structures [47]. Consequently, printability in DIW is governed primarily by molecular architecture rather than by external rheology modifiers. Following extrusion and subsequent photocuring, DIW‐printed specimens display good mechanical performance, as evidenced by the stress–strain response in Figure 4d. The cured materials exhibit an elastic modulus of 8 ± 0.25 MPa together with high elastomeric extensibility (>300%), indicating that photocrosslinking stabilizes the printed macromolecular architecture while retaining segmental mobility. Notably, this stiffness exceeds previously reported depolymerizable δ‐lactone systems, which typically display moduli on the order of ∼100 kPa, representing an improvement of approximately two orders of magnitude in modulus while maintaining substantial ductility. Mechanical robustness under repeated deformation is further evaluated by cyclic tensile testing between 20% and 120% applied strain over more than 100 loading–unloading cycles, which showed stable load responses without noticeable degradation across cycles, as detailed in Figure S23 of the Supporting Information. These results demonstrate that chemically recyclable resins can achieve mechanically robust performance suitable for load‐bearing soft structures without incorporation of non‐depolymerizable components. The high shape fidelity of DIW‐printed structures is demonstrated in Figure 4e, where multilayer architectures maintain well‐defined features and vertical walls immediately after printing and subsequent photocuring, confirming that the room‐temperature rheological response of the uncured inks is sufficient to support complex geometries without collapse or flow‐induced distortion. Beyond structural printing, the DIW ink platform readily accommodates functional additives without compromising extrusion or curing behavior. As shown in Figure 4f–i, incorporation of 1 wt.% multiwalled carbon nanotubes (MWCNTs) yields electrically conductive inks that can be printed with high shape fidelity and subsequently photocured to form stable conductive architectures. The resulting printed features exhibit continuous conductive pathways, enabling electrical activation of printed elements, as demonstrated by illumination under an applied potential of 5 V. Importantly, functionality is introduced through physical filler incorporation rather than chemical modification of the polyester backbone, preserving the depolymerizable network chemistry and underscoring the versatility of the DIW‐compatible resin system for multifunctional, recyclable printed devices.

In contrast to DIW processing at room temperature, DLP printing requires a low‐viscosity, homogeneous resin capable of rapid recoating and uniform light exposure [48, 49]. Because the formulation contains semicrystalline δ0‐rich segments that elevate viscosity under ambient conditions, temperature is used as a controlled parameter to modulate flow behavior. As shown in Figure 4j, increasing the resin temperature enhances molecular mobility and simultaneously melts the δ0 microcrystalline domains, leading to a significant decrease in viscosity across the shear‐rate range relevant to DLP 3D printing. Under these conditions, the resin transitions from a physically structured melt to a uniform, low‐viscosity photocurable liquid, placing its flow behavior within the processing window required for DLP 3D printing. This thermally induced viscosity tuning enables the same depolymerizable polyester chemistry employed for DIW to be adapted for DLP printing while preserving the underlying network chemistry and crosslinking strategy. To enable DLP printing under elevated‐temperature conditions, a custom heated vat system is used to maintain consistent resin flow during printing. The system incorporates temperature‐controlled hot‐water tubing integrated within the vat, providing uniform heating of the resin throughout operation, which can heat the resin up to 60°C. Continuous thermal regulation prevents viscosity buildup during printing and ensures reliable resin recoating under conditions required to melt δ0‐rich microcrystalline domains. A schematic and photograph of the heated vat system are provided in Figure S24, and further details of the design and operation have been reported in our previous publication [7]. DLP‐printed parts produced from the thermally conditioned resin formulations exhibit mechanically stable elastomeric behavior after photocuring, as summarized in Figure 4k. The resulting materials show an elastic modulus of 5.5 ± 0.8 MPa, indicating that effective network formation is achieved despite the lower molecular weight of the precursors used for vat‐based processing. The combination of high extensibility and smooth stress evolution confirms that elevated‐temperature printing does not compromise mechanical integrity. Durability under repeated deformation is assessed through cyclic tensile loading between 20% and 120% applied strain for more than 100 cycles, during which consistent load responses are maintained without measurable degradation, as illustrated in Figure S25. The capability of the thermally conditioned resin system to support high‐resolution DLP 3D printing is demonstrated in Figure 4l, where DLP‐printed objects exhibit well‐defined features and smooth surfaces across complex geometries. The printed structures maintain dimensional accuracy and sharp edges, indicating that uniform resin flow and controlled photopolymerization are achieved under elevated‐temperature printing conditions. These results confirm that temperature‐enabled viscosity reduction is sufficient to ensure reliable recoating and layer formation during DLP printing without sacrificing geometric fidelity. In addition to structural printing, the DLP resin platform can be extended to ionic sensing applications through incorporation of lithium bis(trifluoromethanesulfonyl)imide (LiTFSI). As schematically illustrated in Figure 4m, addition of 10 wt.% LiTFSI to the photocurable formulation yields a crosslinked polyester network containing mobile ions, enabling the conceptual design of deformable ionic strain sensors within the same depolymerizable material framework [50]. The functional performance of this approach is evaluated in Figure 4n, where DLP‐printed specimens exhibit a reproducible, strain‐dependent electrical response under mechanical deformation. This behavior confirms that ionic transport within the photocured network remains responsive to applied strain, demonstrating the feasibility of fabricating recyclable, DLP‐printed functional materials for various applications.

### 2.4 Thermoplastic 3D Printing (FFF)

**FIGURE 5:**
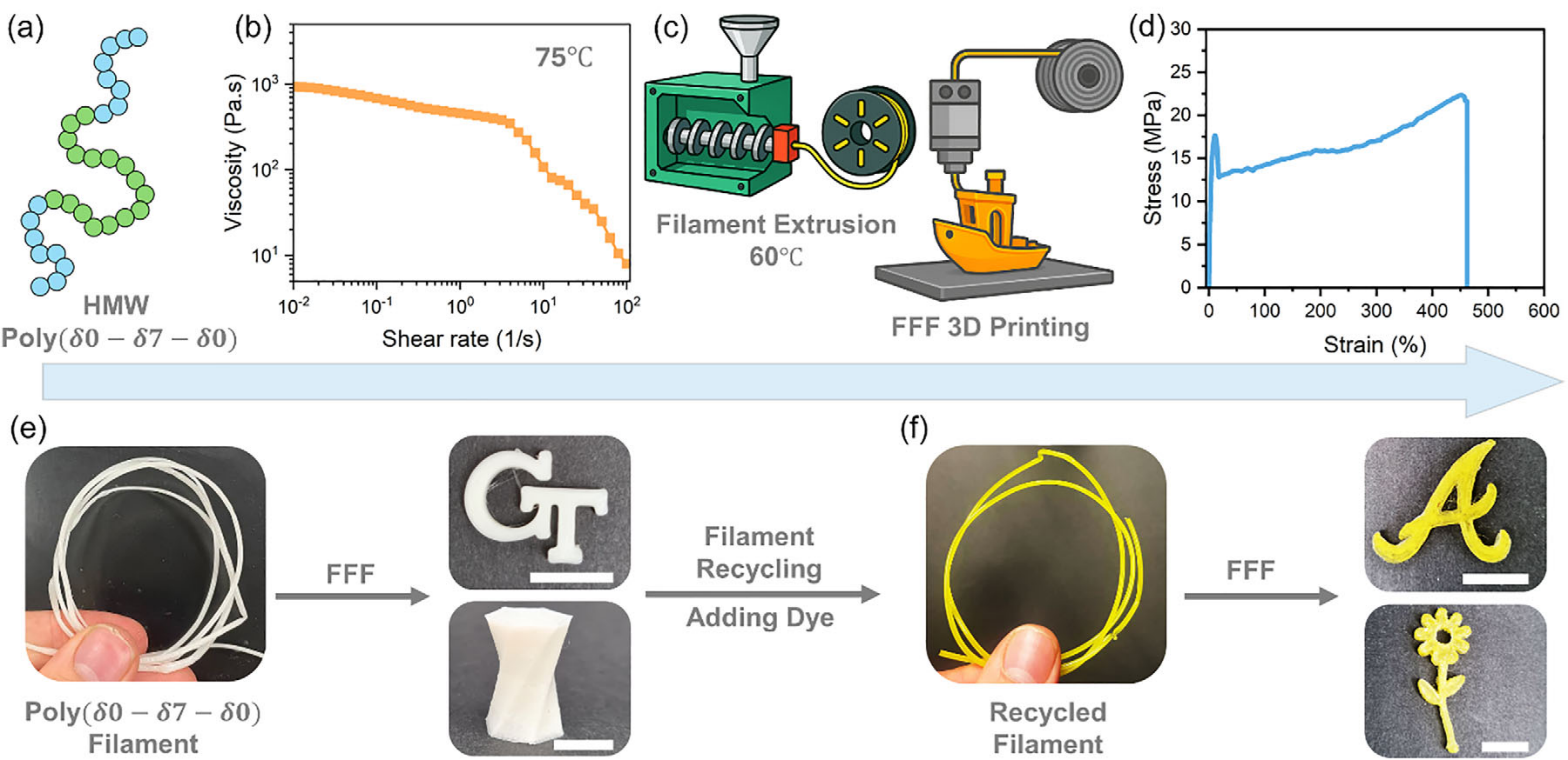
Overview illustration of incorporation of poly(δ0–δ7–δ0) as a precursor for FFF 3D printing. (a) Schematic representation of synthesis of HMW poly(δ0–δ7–δ0). (b) Viscosity‐shear rate graph of the synthesized polymer at 75°C showing shear‐thinning behavior. (c) Schematic illustration filament fabrication and FFF 3D printing of the synthesize copolymer. (d) Tensile stress‐strain graph of HMW poly(δ0–δ7–δ0) copolymer showing thermoplastic like behavior. (e) Extruded filament and 3D printed objects using as‐synthesized material. (f) Recycled filament and 3D printed object using recycled and colored filament. All scale bars are 10 mm.

To extend the δ‐lactone platform to melt‐based additive manufacturing, the copolymer architecture is deliberately shifted into a fully thermoplastic regime through a substantial increase in molecular weight (Figure 5a). Both the amorphous δ7 midblock and the semicrystalline δ0 hard segments are targeted at high degrees of polymerization to ensure sufficient melt strength and mechanical integrity under extrusion conditions [51]. The poly(δ7) midblock is synthesized to a number‐average molecular weight of approximately 20 kg.mol^−^^1^, followed by chain extension with poly(δ0) to yield segmented copolymers with total molecular weights exceeding 60 kg.mol^−^^1^, which is above the critical entanglement threshold required for stable thermoplastic processing [52]. At these molecular weights, without necessity of post functionalization, the combination of extensive chain entanglement within the amorphous domains and physical reinforcement from dispersed crystalline δ0 segments produces a mechanically robust melt capable of sustaining the tensile, shear, and compressive stresses encountered during filament extrusion and fused filament fabrication (FFF) 3D printing. This architecture preserves the depolymerizable polyester backbone while imparting the rheological and mechanical characteristics necessary for reliable thermoplastic 3D printing. As shown in Figure 5b, rheological characterization performed at 75°C confirms that the high‐molecular‐weight copolymer exhibits melt behavior well suited FFF [53]. The material displays a high zero‐shear viscosity at low shear rates, indicative of extensive chain entanglement and sufficient melt strength to prevent filament sagging or die swell during extrusion. With increasing shear rate, the viscosity decreases markedly, reflecting pronounced shear‐thinning behavior characteristic of entangled thermoplastic melts used in FFF 3D printing. At this processing temperature, melting of the semicrystalline δ0 domains effectively lowers the melt viscosity into the range required for smooth filament extrusion, while the δ7‐rich amorphous matrix undergoes shear‐induced chain alignment that enables facile flow under load yet rapid elastic recovery upon deposition. Together, this combination of thermally activated softening and shear‐responsive flow behavior places the copolymer squarely within the rheological window of commercially viable FFF thermoplastics.

Building on this favorable melt rheology, the copolymer is processed into continuous filaments using a low‐temperature extrusion protocol, as illustrated in Figure 5c. Filament fabrication at 60°C enables sufficient melt flow while preserving the chemical integrity of the depolymerizable polyester backbone. The resulting filaments can be remelted and printed using a standard FFF setup, demonstrating that the same δ‐lactone chemistry previously configured for DIW and DLP can also be translated into a mechanically robust, high molecular weight thermoplastic for extrusion‐based 3D printing. This extension of the platform from photocurable formulations to reinforced thermoplastic feedstocks is achieved without sacrificing chemical recyclability, and the printed materials exhibit enhanced mechanical performance, as illustrated in Figure 5d. The sample demonstrated a Young's modulus of 430 ±  12 MPa, while maintaining an elongation at break above 450%. The high molecular weight copolymer can be readily melt‐processed into continuous filaments with a relatively uniform diameter, confirming its suitability for extrusion‐based printing (Figure 5e). The filaments retain sufficient flexibility for handling and spooling while maintaining the melt strength required for stable feeding through an FFF nozzle. When printed, the material yields well‐defined geometries, including the GT emblem and a twisted hexagonal column, with smooth strand deposition and clear feature boundaries as shown in Figure 5e. This printing fidelity is consistent with rapid crystallization of the δ0 segments during cooling, which stabilizes deposited filaments and preserves structural definition. The thermoplastic character of the copolymer further enables straightforward reprocessing (Figure 5f). Remelting and re‐extrusion of previously printed material yields filaments with comparable dimensional uniformity and handling characteristics, indicating minimal degradation during thermal cycling. Introduction of a colorant during remelting produces uniformly dyed filaments that can be reprinted into complex geometries, with feature definition comparable to the original prints. These results demonstrate that the copolymer can undergo multiple melt processing and printing cycles while maintaining print fidelity. These observations further suggest that the synthesized copolymer is compatible with the dyes, allowing them to be uniformly distributed within the filament during extrusion.

### 2.5 Recycling and Reprocessability

**FIGURE 6:**
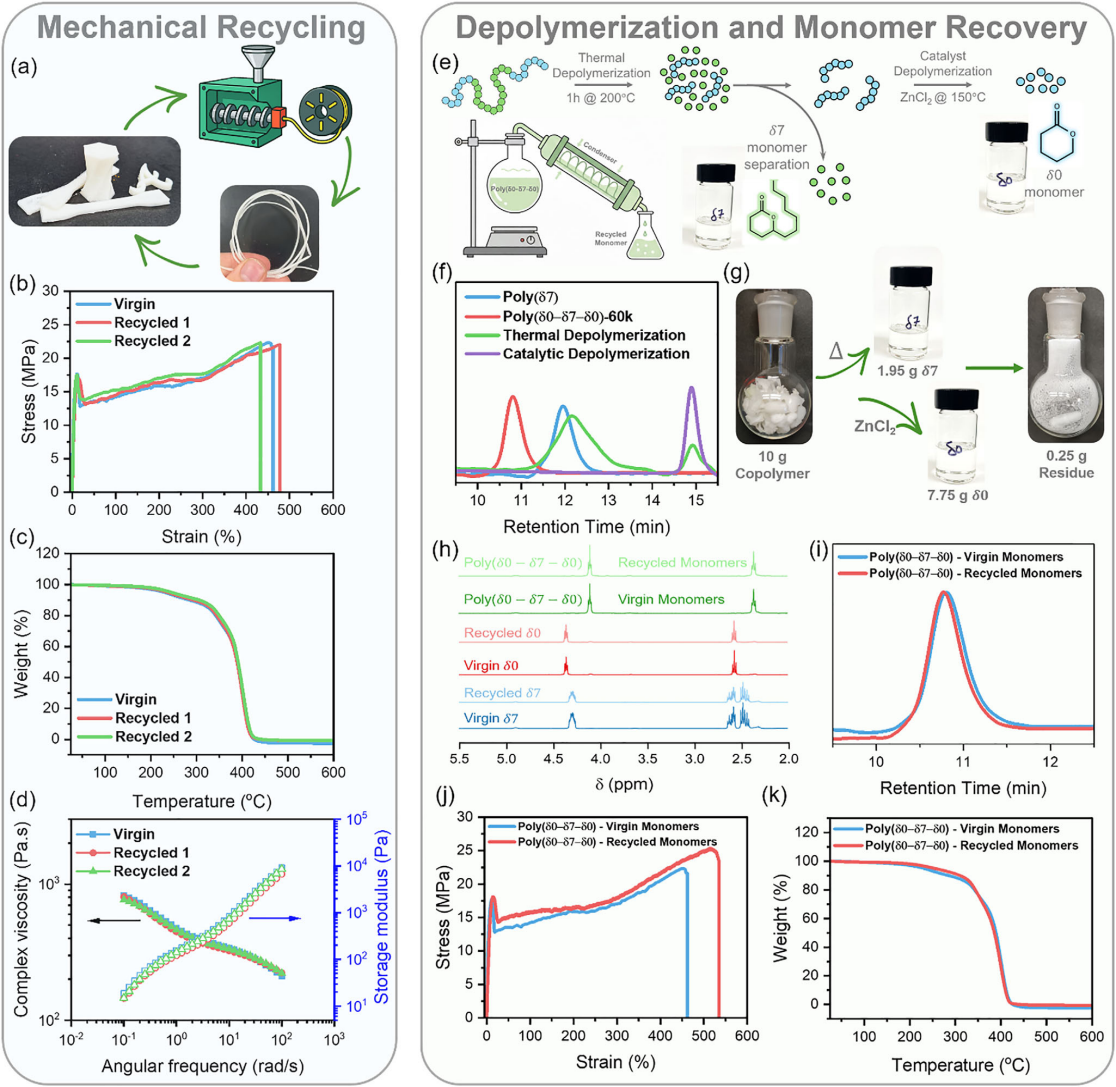
Overview of the mechanical recyclability and chemical depolymerization of the poly(δ0‐δ7‐δ0) copolymers. (a) Schematic workflow illustrating the mechanical recycling process for the HMW poly(δ0‐δ7‐δ0) copolymer used in FFF 3D printing. (b) Tensile stress‐strain curves of the virgin copolymer and materials recovered after two mechanical recycling cycles. (c) TGA profiles showing identical thermal degradation behavior for virgin and recycled samples. (d) Frequency sweep demonstrating overlapping complex viscosity and storage modulus for virgin and recycled materials. (e) Schematic workflow representation of two‐stage thermal‐catalytic depolymerization and monomer separation of poly(δ0‐δ7‐δ0) copolymer. (f) Depolymerization of 10 g copolymer in a round‐bottom flask yielding ∼95% of initial monomer used. (g) Retention time of copolymer going through two‐stage depolymerization, the copolymer experiences thermal depolymerization at 200°C followed by catalytic depolymerization that yield fully depolymerized monomers. (h) Comparison of retention time of copolymer synthesized by virgin monomer and recycled monomers. (i) Comparison of ^1^H‐NMR spectra of virgin and recycled δ7 and δ0 monomer along with the copolymer synthesized by virgin and recycled monomers. (j) Tensile stress‐strain curves of polymer synthesized by virgin and recycled monomer. (k) TGA profiles showing identical thermal degradation behavior for copolymer synthesized by virgin and recycled monomer.

To assess the recyclability of the developed polymeric system, we perform a combined evaluation of mechanical reprocessing and chemical depolymerization pathways (Figure 6). These routes represent distinct yet complementary strategies for material recovery: mechanical reprocessing enables direct reuse of the polymer through melt‐based reshaping while preserving its macromolecular architecture, whereas chemical depolymerization allows recovery of the constituent δ‐lactone monomers for re‐synthesis. Considering both pathways provides a comprehensive assessment of the material's ability to undergo repeated processing cycles, either at the polymer or monomer level, without degradation of key properties or chemical fidelity. The following sections analyze each pathway in detail, linking processing history to mechanical, thermal, rheological, and compositional outcomes.

#### 2.5.1 Mechanical Recycling

The high‐molecular‐weight δ‐lactone copolymer exhibits excellent compatibility with mechanical recycling, retaining both structural integrity and functional performance over multiple reprocessing cycles. As illustrated schematically in Figure 6a, printed components are mechanically grounded, remelted at 60°C, and re‐extruded to regenerate filaments suitable for subsequent FFF 3D printing. The reprocessing does not require any solvent. This reprocessability arises from the intrinsic thermoplastic nature of the segmented architecture. Mechanical testing confirms that repeated melt reprocessing does not compromise mechanical performance. The stress–strain curves in Figure 6b show substantial overlap between virgin and twice‐recycled specimens, with no measurable changes in yield strength, elongation at break, or overall toughness. This invariance reflects the thermal stability of the polyester backbone and the reproducible reformation of the crystalline–amorphous phase structure during each processing cycle. Thermal and rheological analyses further corroborate the robustness of the material under mechanical recycling. Thermogravimetric profiles (Figure 6c) display indistinguishable degradation onset temperatures and mass‐loss behavior for virgin and recycled samples, indicating the absence of thermal degradation or accumulation of low‐molecular‐weight species during reprocessing. Complete thermogravimetric and derivative mass‐loss profiles in the (Figures S26 and S27) reveal no additional degradation events, indicating equivalent thermal stability. Similarly, frequency sweep measurements as shown in Figure 6d reveal nearly identical complex viscosity and storage modulus across all samples, demonstrating that chain entanglement density and crystallization‐mediated elasticity are preserved. These results establish that the δ‐lactone copolymer maintains its melt rheology, thermal stability, and mechanical performance through repeated mechanical recycling. It is worth noting that, because mechanical recycling is performed at a relatively low temperature, no visible color change is observed in the filament after 3 recycling cycles.

#### 2.5.2 Chemical Depolymerization and Monomer Regeneration

Beyond melt reprocessing, the δ‐lactone copolymer is engineered to enable chemical recycling through controlled depolymerization and selective monomer recovery. As illustrated in Figure 6e, this process exploits the contrast in ceiling temperatures of the δ7 and δ0 repeat units to achieve stepwise depolymerization and separation. Owing to the relatively low ceiling temperature of δ7, thermal treatment at a moderate temperature (200°C for 1 h) induces preferential depolymerization of the δ7‐rich segments, while the higher‐ceiling‐temperature δ0 units remain chemically stable under these conditions. To minimize any risk of δ0 depolymerization during monomer isolation, the system temperature is subsequently reduced to 150°C, which is still above the boiling point of δ7 (∼130°C), allowing efficient distillation and recovery of the depolymerized δ7 monomer. Following removal of the δ7 fraction, catalytic depolymerization of the remaining δ0‐rich residue is initiated by addition of ZnCl_2_ (2 wt.%), a known Lewis acid catalyst for δ‐lactone depolymerization [54, 55, 56]. Continued heating at 150°C for an additional 3 h, under distillation conditions, enables efficient unzipping of the δ0 segments and recovery of the corresponding monomer. This sequential thermal–catalytic strategy allows both monomers to be recovered under relatively mild conditions while preserving monomer purity and minimizing side reactions. Further insight into the depolymerization mechanism is provided by GPC analysis (Figure 6f), which tracks the molecular weight evolution throughout copolymerization and sequential depolymerization. The poly(δ7) homopolymer macroinitiator exhibits a number‐average molecular weight (M_n_) of approximately 20 kg mol^−^^1^, which increases to ∼60 kg.mol^−^^1^ following chain extension with δ0. Given the bifunctional nature of the macroinitiator, this increase is consistent with the growth of δ0 segments from both chain ends, yielding arms of comparable length. Upon thermal depolymerization, a new low–retention‐time peak emerges at ∼15 min, corresponding to depolymerized δ7 monomer, while the main polymer peak shifts to ∼18.5 kg mol^−^^1^ with a modestly broadened distribution. This behavior indicates selective depolymerization of the δ7 segments while preserving the δ0‐rich backbone. Subsequent catalytic depolymerization results in the complete disappearance of high‐molecular‐weight species and the appearance of a single sharp peak at ∼15 min, consistent with full conversion to monomeric species. These results provide direct molecular‐level evidence that depolymerization proceeds sequentially, validating the selective unzipping strategy enabled by the distinct ceiling temperatures of the two lactone components. The efficiency of the depolymerization and recovery process is reflected in the mass balance shown in Figure 6g. Starting from 10 g of copolymer, sequential depolymerization yields 1.95 g of recovered δ7 monomer and 7.75 g of recovered δ0 monomer, with only a small residual fraction (0.25 g) remaining after completion of the process. The low residue content indicates a high degree of backbone conversion and minimal formation of non‐volatile byproducts, underscoring the effectiveness of the stepwise depolymerization strategy in enabling near‐quantitative monomer recovery. ^1^H NMR analysis of the post‐distillation residue further confirms the high depolymerization efficiency, showing an approximately 96.5% reduction in the characteristic copolymer peak intensities relative to the original copolymer spectrum (Figure S28). This result agrees well with the monomer recovery efficiency determined from the mass balance. To assess whether network formation interferes with chemical recyclability, depolymerization is also performed on crosslinked samples fabricated by DLP and DIW printing using the same sequential depolymerization protocol. The recycling efficiencies of FFF, DLP, and DIW samples are summarized in Figure S29. While the uncrosslinked FFF samples exhibit a depolymerization efficiency of approximately 97%, the crosslinked DLP and DIW samples retain similarly high efficiencies of 94% and 95%, respectively. These small differences likely arise primarily from the formulation requirements associated with each printing method. Specifically, the FFF samples are processed from unmodified thermoplastic copolymers and therefore contain the highest fraction of recoverable δ‐lactone‐derived polyester segments. In contrast, the DIW and DLP formulations require TMSPMA‐functionalized copolymers and Poly(δ7)‐2HEA to enable photocrosslinking and printability, which slightly reduces the relative fraction of recoverable polyester in the final materials. The slightly higher efficiency of DIW compared with DLP is also consistent with this compositional explanation, as the DIW formulation contains a higher molecular weight Poly(δ0‐δ7‐δ0) copolymer and a lower amount of Poly(δ7)‐2HEA than the DLP formulation. In addition, partial consumption or immobilization of depolymerization‐active chain ends during network formation may modestly reduce the population of chain termini available to initiate unzipping. Nevertheless, the high recovery efficiencies demonstrate that covalent crosslinking does not significantly impede depolymerization, confirming the compatibility of the polymer network with chemical recycling across multiple additive manufacturing modalities. In addition to monomer recovery, functional additives incorporated in DIW‐ and DLP‐printed materials are also 100% recovered during chemical depolymerization and reused without loss of performance. Both MWCNT and LiTFSI are isolated from the depolymerized mixtures and reintroduced into fresh formulations. Electrical measurements of samples printed using the recycled additives (Figure S30a,b) show resistances comparable to those of materials prepared with virgin additives, indicating minimal degradation or loss of conductivity. It is worth mentioning that chemical recycling experiments are carried out directly using the structures fabricated by DIW, DLP, and FFF printing. Prior to depolymerization, the samples are only manually chopped into smaller pieces to fit into the round‐bottom flask and facilitate stirring. No grinding, dissolution, solvent addition, or chemical pretreatment is required, and monomers are recovered directly under distillation conditions.

^1^H NMR spectroscopy is used to evaluate the chemical fidelity of the recovered monomers and polymers following depolymerization, as shown in Figure 6h. The spectra of recycled δ7 and δ0 monomers show complete overlap with those of their virgin counterparts, with no detectable additional resonances or peak broadening, indicating the absence of side reactions or chemical degradation during the depolymerization process. To further verify monomer integrity, copolymers are resynthesized from the recycled monomers and compared directly with copolymers prepared from virgin feedstocks. The resulting ^1^H NMR spectra are indistinguishable, confirming that monomer recovery proceeds without alteration of functional groups or backbone structure. To assess whether chemical recycling preserves the material's macroscopic performance, the physical properties of copolymers synthesized from recycled monomers are directly compared with those of copolymers prepared from virgin monomers. GPC analysis, shown in Figure 6i, reveals nearly identical retention times for the two materials, indicating comparable molecular weight distributions following re‐polymerization. Mechanical testing in Figure 6j further confirms property retention, with stress–strain responses of recycled‐monomer copolymers closely matching those of the virgin material. Thermal stability is likewise preserved, as thermogravimetric analysis presented in Figure 6k shows indistinguishable degradation profiles for both samples. Full thermogravimetric and derivative weight‐loss data provided in the Supporting Information (Figures S22 and S31) show no additional degradation features, confirming comparable thermal stability. These results demonstrate that monomer recovery and reuse do not compromise molecular architecture or bulk material performance, validating chemical recycling as an effective route for regenerating high‐quality copolymers.

To further demonstrate the full circularity of this material platform, monomers recovered through chemical recycling are reused to synthesize both the Poly(δ0‐δ7‐δ0)‐DMA crosslinker and the Poly(δ7)‐2HEA reactive macromonomer. The recycled‐monomer‐derived polymers are prepared using the same polymerization and functionalization procedures used for the virgin‐monomer‐derived materials (state in above sections). The resulting resin exhibits photocuring characteristics comparable to the original DLP resin, enabling successful fabrication of a BCC lattice structure by DLP printing (Figure S32). These results demonstrate that the recovered monomers can be directly reintroduced into the resin synthesis workflow without compromising resin processability or print quality.

## 3 Conclusion

This work establishes a δ‐lactone materials platform capable of spanning a wide spectrum of polymer‐based additive manufacturing while retaining complete end‐of‐life circularity. By strategically manipulating molecular weight and segmental architecture, the same depolymerizable chemistry can be configured as a photocurable resin for DIW and DLP or as a high‐molecular‐weight thermoplastic for FFF. The resulting materials exhibit predictable rheology and excellent print fidelity across all three processes, demonstrating that targeted molecular design can reconcile the distinct requirements of filament‐, ink‐, and resin‐based 3D printing. The copolymers can be fully depolymerized back to their δ7 and δ0 monomers through a two‐stage thermal–catalytic process, achieving high monomer recovery efficiencies of 94% for DLP‐printed samples, 95% for DIW‐printed samples, and 97% for FFF‐printed materials. The recovered monomers exhibit chromatographic and spectroscopic purity indistinguishable from virgin feedstocks and can be repolymerized into new materials with identical mechanical and thermal properties. This molecular‐level recyclability distinguishes the platform from conventional polyesters and establishes a complete chemical circularity cycle under mild and scalable conditions. Moreover, the high MW copolymers synthesized for FFF 3D printing, can be mechanically reprocessed several times. Thermal, rheological, and mechanical measurements all confirm that the polymer maintains its structural and functional integrity upon repeated reprocessing, enabling a closed‐loop mechanical recycling route without loss of performance. Importantly, copolymerization enhances the mechanical properties by up to three orders of magnitude compared to previously reported depolymerizable lactone‐based systems, demonstrating that a single polymer backbone, designed with controlled segmental chemistry and tunable molecular weight, can meet the disparate demands of high‐resolution printing, robust mechanical performance, and fully circular end‐of‐life recovery. This work introduces a new design paradigm for high‐performance, chemically recyclable polymers tailored for additive manufacturing, offering a versatile blueprint for next‐generation sustainable materials systems.

## 4 Experimental Section

### 4.1 Materials

δ‐Dodecalactone (δ7), δ‐Valerolactone (δ0) diphenyl phosphate (DPP), Ethylene Glycol (EG), 2‐hydroxyethyl acrylate (2HEA), Isobornyl Acrylate (IBOA), Butyl Acrylate (BA), calcium chloride, lithium bis(trifluoromethanesulfonyl)imide (LiTFSI), Phenylbis(2,4,6‐trimethylbenzoyl)phosphine oxide (PI819), Zinc chloride (ZnCl_2_), 3‐(trimethoxysilyl)propyl methacrylate (TMSPMA), Sudan I and all the solvent used were purchased from Sigma–Aldrich (St. Louis, MO, USA). Oil soluble dye Solvent Blue 104 was from Clariant Corp. (Frankfurt, Germany) and Solvent green 5 was from Orichem International Ltd. (Hangzhou, Zhejiang, China). Aliphatic urethane diacrylate (AUD, Ebecryl 8413) was from Allnex (Alpharetta, GA, USA).

### 4.2 Material Synthesis

All glassware, stir bars, and 50 mL reaction vessels were dried overnight in a 110°C vacuum oven and transferred into a nitrogen‐filled glovebox prior to use. Monomers and initiators were dried over molecular sieves and stored under nitrogen. Polymerizations were performed in dried, nitrogen‐purged reactors sealed with PTFE‐lined caps. Diphenyl phosphate (DPP) was used as catalyst at a monomer:catalyst ratio of 100:1 unless otherwise noted. All reactions were stirred under nitrogen atmosphere, and conversion was monitored by periodically withdrawing aliquots, quenching to air, and analyzing by ^1^H NMR (CDCl_3_, 1% internal standard). Molecular weights and dispersities were determined by GPC (CHCl_3_ eluent, polystyrene calibration standards). Final polymers were dried under vacuum prior to characterization.

Poly(δ7) homopolymers were synthesized by ROP of δ‐dodecalactone initiated from either ethylene glycol (EG) or 2‐hydroxyethyl acrylate (2‐HEA), following the general polymerization conditions described above. The monomer‐to‐initiator ratio was selected to target the desired number‐average molecular weight (Table 1). Inside the glovebox, monomer, initiator, and DPP catalyst were combined to the desired monomer concentration and sealed. Reactions were carried out at room temperature under nitrogen for 24–48 h before workup and characterization as described in the general protocol. poly(δ0–δ7–δ0) block copolymers were prepared by sequential ROP using (unwashed) Poly(δ7) as a macroinitiator, following the general polymerization conditions. In the glovebox, Poly(δ7), and δ0 and, was added to dried glass reactors; anhydrous CHCl_3_ was used as diluent to achieve a monomer concentration of 2–3 m. The monomer‐to‐macroinitiator ratio determined the targeted block length. Sealed reactors were placed in a 70°C oil bath and stirred for 10–36 h depending on composition. For end‐group functionalization, hydroxyl‐terminated poly(δ7) and poly(δ0–δ7–δ0) polymers were reacted with 2.2 equiv. of TMSPMA per polymer chain, corresponding to 1.1 equiv. per terminal hydroxyl group, under bulk conditions without the use of solvent. The reaction was carried out at 80°C for 5 h to introduce terminal methacrylate groups.

After polymerization reaction as well as functionalization process, the polymer products were purified by repeated precipitation in methanol. Briefly, the reaction mixtures were precipitated into methanol to remove residual unreacted monomers and initiator species, while the polymers remain insoluble and were collected as precipitates. This purification process was repeated three times, and the purified polymers were dried in a vacuum oven at 50°C overnight before further use.

### 4.3 Material Characterization

The Molecular weight of the synthesized polymer samples were analyzed using a Tosoh Bioscience EcoSEC Elite‐WS HLC‐8420GPC system with TSKgel SuperHZ‐L columns. The samples with concentration 1 mg/mL were dissolved in CHCl_3_ solution containing 0.25 wt.% TEA and stirred for 2 h before doing the GPC measurement. For GPC experiments, three samples were tested for each polymer. NMR(nuclear magnetic resonance) spectra were recorded on Bruker Avance 400 MHz instrument. The NMR samples were prepared with concentration of 10 mg/mL in CDCl_3_ solution with 1 wt.% internal standard. FTIR (fourier‐transform infrared spectroscopy, Nicolet 6700 spectrometer, Thermo Fisher Scientific) was measured in absorbance model from 500 to 4000cm^−1^. TGA (thermogravimetric analysis, Mettler Toledo TGA2 STAR System) was performed under nitrogen from 25°C to 600°C with a heating rate of 10°C/min. Three independent TGA measurements were performed for each polymer to assess the reproducibility of the thermal degradation profiles. Rheology tests were performed with a Discovery HR‐2 (TA Instruments, New Castle, DE, USA) using a 20 mm diameter plate. The samples were kept in the test temperature for 2 min before starting the measurement. In flow‐sweep test, the samples are subjected to shear rate from 0.01 to 100 1/s. For frequency‐sweep tests, angular frequency was changed from 100 to 0.1 rad/s with strain of 1%. For photo‐rheology test, a UV light base (385 nm, 25 mW/cm^2^) was used and the storage and loss moduli were monitored over time. The uniaxial tension tests and load‐unload tests were conducted with a universal test machine (Insight 10, MTS Systems Corp.). Tensile specimens for the DIW, DLP, and FFF methods were fabricated using the corresponding 3D printing technique. In all cases, tensile bars were prepared following the ASTM D638 Type IV standard [57]. Three tensile measurements were performed for each material to evaluate the reproducibility of the stress–strain response and the resulting mechanical properties. DSC analyses were measured on a DSC system (Discovery DSC 250, TA Instruments, New Castle, DE, USA) with heating rate of 5°C min^−1^. The resistance of the conducive samples was measured by a Keithley 2100 Series: 6.5 Digit USB Multimeter.

### 4.4 3D Printing

#### 4.4.1 DIW

For DIW 3D‐printing ink preparation, the Poly(δ0‐δ7‐δ0)‐30k copolymer and Poly(δ7)‐2HEA‐4k were blended at a 75:25 weight ratio, and 1 wt.% of PI819 photoinitiator was incorporated into the mixture. After mixing thoroughly, the ink was transferred into a 10 cc syringe (Nordson EFD, East Providence, RI, USA) and degassed by centrifugation at 4000 rpm for 15 min. The loaded syringe was mounted onto a customized DIW 3D‐printing platform, where extrusion was controlled by a high‐precision fluid dispenser (Ultimus I, Nordson EFD, USA) operating at a constant pressure of 15 psi under ambient conditions. Printing was carried out at a linear speed of 5 mm.s^−1^ through an 18‐gauge nozzle, with each deposited layer photocured for 10s using a UV flashlight (20 mW.cm^−2^).

#### 4.4.2 DLP

For DLP 3D‐printing resin preparation, the Poly(δ0‐δ7‐δ0)‐17k copolymer and Poly(δ7)‐2HEA‐2k were blended at a 50:50 weight ratio, and 1 wt.% PI819 photoinitiator along with 0.1 wt.% of a photoabsorber (Sudan I, Clariant Solvaperm Blue 2B‐CN, or Solvent green 5) was added. Due to the high viscosity of the formulation, the mixture was heated to 60°C and stirred for 1 h to ensure complete homogenization. 3D printing was conducted using a bottom‐up DLP printer equipped with a 385 nm UV‐LED projector (PRO4500, Wintech Digital Systems Technology Corp., Carlsbad, CA, USA) and a linear translation stage (LTS150, Thorlabs, Newton, NJ, USA). A custom resin vat incorporating an oxygen‐permeable Teflon AF‐2400 window (Vici Metronics Inc., Poulsbo, WA, USA) was used during printing. Samples were fabricated with a 50 µm layer thickness and an exposure time of 5s per layer. To address resin viscosity limitations, the DLP system was integrated with a custom heated‐vat assembly designed and fabricated in‐house. The vat temperature was maintained by circulating hot water through tubing embedded along the vat's inner perimeter, driven by a DC pump connected to a heated water reservoir. Additional details regarding the heated‐vat design and operation were provided in our previous work [7].

#### 4.4.3 FFF

For FFF 3D‐printing, the high–molecular‐weight Poly(δ0‐δ7‐δ0)‐60k copolymer was used to fabricate the printable filament. The polymer was first melted by heating to 70°C for 30 min inside a stainless‐steel syringe barrel, followed by controlled cooling to 50°C prior to extrusion. Filaments with a diameter of 1.6–1.8 mm were produced and subsequently employed for printing. FFF 3D printing was performed using an Ender 5 system equipped with a direct‐drive extruder. Printing was conducted at a nozzle temperature of 75°C, a layer height of 0.2 mm, and a linear print speed of 50 mm.s^−^^1^, with the build platform maintained at 25°C.

## Conflicts of Interest

The authors declare no conflicts of interest.

## Supporting information




**Supporting File**: adma73986‐sup‐0001‐SuppMat.docx.

## Data Availability

The data that supports the findings of this study are available in the supplementary material of this article.
