# Supplementary material for: Mechanically Robust and Depolymerizable Polyesters With Near‐Complete Monomer Recovery for Circular Additive Manufacturing
Source: Adv Mater. 2026 Jul 9;38(46):e73986. doi: 10.1002/adma.73986 (PMC13486233; doi:10.1002/adma.73986)
Supplement: Supplementary file 1 [file ADMA-38-e73986-s001.docx]

Supplementary Materials for

**Mechanically Robust and Depolymerizable Polyesters with Near-Complete Monomer Recovery for Circular Additive Manufacturing**

Farzad Gholami^1^, Rampi Ramprasad^1^ H. Jerry Qi^1,2^

^1^School of Materials Science and Engineering, Georgia Institute of Technology, Atlanta, GA, USA

^2^George W. Woodruff School of Mechanical Engineering, Georgia Institute of Technology, Atlanta, GA, USA

* Corresponding author, [qih@me.gatech.edu](mailto:qih@me.gatech.edu)

**The PDF file includes:**

[**Figure S1.** ^1^H NMR spectrum of poly(δ7) homopolymer grafted on 2-HEA as initiator with detailed peak assignment.](#_Toc231557386)

[**Figure S2.** ^1^H NMR of $\delta$-Dodecalactone monomer in CDCl_3_ in presence of 1 wt.% internal standard.](#_Toc231557387)

[**Figure S3.** ^1^H NMR of Poly($\delta$7) polymer after 2h of reaction in CDCl_3_ in presence of 1 wt.% internal standard.](#_Toc231557388)

[**Figure S4.** ^1^H NMR of Poly($\delta$7) polymer after 6h of reaction in CDCl_3_ in presence of 1 wt.% internal standard.](#_Toc231557389)

[**Figure S5.** ^1^H NMR of Poly($\delta$7) polymer after 8h of reaction in CDCl_3_ in presence of 1 wt.% internal standard.](#_Toc231557390)

[**Figure S6.** 1H NMR of Poly($\delta$7) polymer after 12h of reaction in CDCl3 in presence of 1 wt.% internal standard.](#_Toc231557391)

[**Figure S7.** ^1^H NMR of Poly($\delta$7) polymer after 22h of reaction in CDCl_3_ in presence of 1 wt.% internal standard.](#_Toc231557392)

[**Figure S8.** ^1^H NMR of Poly($\delta$7) polymer after 36h of reaction in CDCl_3_ in presence of 1 wt.% internal standard.](#_Toc231557393)

[**Figure S9.** ^1^H NMR of Poly(δ0-δ7-δ0) copolymer immediately after adding δ0 monomer to the poly(δ7) macroinitiator. The material is dissolved in CDCl_3_ in presence of 1 wt.% internal standard.](#_Toc231557394)

[**Figure S10.** ^1^H NMR of Poly(δ0-δ7-δ0) polymer after 3h of reaction in CDCl_3_ in presence of 1 wt.% internal standard.](#_Toc231557395)

[**Figure S11**. ^1^H NMR of Poly(δ0-δ7-δ0) polymer after 6h of reaction in CDCl_3_ in presence of 1 wt.% internal standard.](#_Toc231557396)

[**Figure S12.** ^1^H NMR of Poly(δ0-δ7-δ0) polymer after 10h of reaction in CDCl_3_ in presence of 1 wt.% internal standard.](#_Toc231557397)

[**Figure S13.** ^1^H NMR of Poly(δ0-δ7-δ0) polymer after 18 h of reaction in CDCl_3_ in presence of 1 wt.% internal standard.](#_Toc231557398)

[**Figure S14.** ^1^H NMR of Poly(δ0-δ7-δ0) polymer after 24h of reaction in CDCl_3_ in presence of 1 wt.% internal standard.](#_Toc231557399)

[**Figure S15.** ^1^H NMR of Poly(δ0-δ7-δ0) polymer after 36h of reaction in CDCl_3_ in presence of 1 wt.% internal standard.](#_Toc231557400)

[**Figure S16**. Time-resolved ^1^H NMR spectra recorded at different reaction times, showing the evolution of monomer and polymer resonances during polymerization of (a) poly(δ7) and (b) poly (δ0-δ7-δ0)](#_Toc231557401)

[**Figure S17.** Monomer conversion as a function of reaction time during sequential homopolymerization and copolymerization.](#_Toc231557402)

[**Figure S18**. ^1^H NMR Spectrum of (a) δ7 monomer, (b) poly(δ7) homopolymer, (c) δ0 monomer, and (d) poly(δ0-δ7-δ0) copolymer with detailed peak assignment. The unassigned peaks of poly(δ0-δ7-δ0) copolymer are for the poly(δ7) mid-block, and their detail can be found in part (b).](#_Toc231557403)

[**Figure S19.** DSC heating traces of poly(δ7) and poly(δ0-δ7-δ0) copolymers with increasing molecular weight, showing a gradual shift in the glass transition temperature (Exo-up graph).](#_Toc231557404)

[**Figure S20.** ^1^H NMR of Poly($\delta$7) and TMSPMA after (a) 2h, (b) 3h, (c) 4h, (d) 5h, and (e) 10h of reaction in CDCl3 in presence of 1 wt.% internal standard.](#_Toc231557405)

[**Figure S21.** (a) Loss modulus and (b) Tan δ graphs for Poly(δ0-δ7-δ0) before and after dimethacrylation and in the presence of 20 wt.% IBOA.](#_Toc231557406)

[**Figure S22.** Tensile stress–strain curves of cured poly(δ7) and poly(δ0–δ7–δ0) networks with different molecular weights formulated with 20 wt.% BA.](#_Toc231557407)

[**Figure S23.** Cyclic loading–unloading stress–strain responses of the DIW printed sample, illustrating repeatable mechanical behavior under successive deformation cycles.](#_Toc231557408)

[**Figure S24.** Schematic and photograph of heated vat system designed to reduce the viscosity of resin for DLP 3D printing.](#_Toc231557409)

[**Figure S25.** Cyclic loading–unloading stress–strain responses of the DLP printed sample, illustrating repeatable mechanical behavior under successive deformation cycles.](#_Toc231557410)

[**Figure S26.** TGA and corresponding derivative weight loss DTG curve of the virging poly(δ0-δ7-δ0)-60k copolymer](#_Toc231557411)

[**Figure S27.** TGA and corresponding derivative weight loss DTG curve of the mechanically recycled poly(δ0-δ7-δ0)-60k copolymer](#_Toc231557412)

[**Figure S28.** Comparison of the ^1^H-NMR spectra with peak integral of (a) the residue remaining after distillation and depolymerization process of poly(δ0-δ7-δ0) and (b) the poly(δ0-δ7-δ0) copolymer right before depolymerization process.](#_Toc231557413)

[**Figure S29.** Recycling efficiency of polymers formulated for DLP, DIW, and FFF.](#_Toc231557414)

[**Figure S30.** Electrical resistance of conductive additives before and after recycling: (a) MWCNTs and (b) LiTFSI.](#_Toc231557415)

[**Figure S31.** TGA and corresponding derivative weight loss DTG curve of the chemically recycled poly(δ0-δ7-δ0)-60k copolymer](#_Toc231557416)

[**Figure S32**. DLP 3D printed lattice using Poly(δ0-δ7-δ0)-DMA + Poly(δ7)-2HEA (50:50). Both copolymer crosslinker and homopolymer macromonomer are synthesized using chemically recycled monomers. The polymerization and functionalization process for both polymers were same as the process for the original polymers synthesized using virgin monomer. Scale bar is 5mm.](#_Toc231557417)

[**Table S1.** Young’s modulus and elongation at break of cured poly(δ7) and poly(δ0–δ7–δ0) networks with different MW formulated with 20 wt.% IBOA](#_Toc231560665)

**Figure S1.** ^1^H NMR spectrum of poly(δ7) homopolymer grafted on 2-HEA as initiator with detailed peak assignment.

**Figure S2.** ^1^H NMR of $\delta$-Dodecalactone monomer in CDCl_3_ in presence of 1 wt.% internal standard.

**Figure S3.** ^1^H NMR of Poly($\delta$7) polymer after 2h of reaction in CDCl_3_ in presence of 1 wt.% internal standard.

**Figure S4.** ^1^H NMR of Poly($\delta$7) polymer after 6h of reaction in CDCl_3_ in presence of 1 wt.% internal standard.

**Figure S5.** ^1^H NMR of Poly($\delta$7) polymer after 8h of reaction in CDCl_3_ in presence of 1 wt.% internal standard.

**Figure S6.** 1H NMR of Poly($\delta$7) polymer after 12h of reaction in CDCl3 in presence of 1 wt.% internal standard.

**Figure S7.** ^1^H NMR of Poly($\delta$7) polymer after 22h of reaction in CDCl_3_ in presence of 1 wt.% internal standard.

**Figure S8.** ^1^H NMR of Poly($\delta$7) polymer after 36h of reaction in CDCl_3_ in presence of 1 wt.% internal standard.

**Figure S9.** ^1^H NMR of Poly(δ0-δ7-δ0) copolymer immediately after adding δ0 monomer to the poly(δ7) macroinitiator. The material is dissolved in CDCl_3_ in presence of 1 wt.% internal standard.

**Figure S10.** ^1^H NMR of Poly(δ0-δ7-δ0) polymer after 3h of reaction in CDCl_3_ in presence of 1 wt.% internal standard.

**Figure S11**. ^1^H NMR of Poly(δ0-δ7-δ0) polymer after 6h of reaction in CDCl_3_ in presence of 1 wt.% internal standard.

**Figure S12.** ^1^H NMR of Poly(δ0-δ7-δ0) polymer after 10h of reaction in CDCl_3_ in presence of 1 wt.% internal standard.

**Figure S13.** ^1^H NMR of Poly(δ0-δ7-δ0) polymer after 18 h of reaction in CDCl_3_ in presence of 1 wt.% internal standard.

**Figure S14.** ^1^H NMR of Poly(δ0-δ7-δ0) polymer after 24h of reaction in CDCl_3_ in presence of 1 wt.% internal standard.

**Figure S15.** ^1^H NMR of Poly(δ0-δ7-δ0) polymer after 36h of reaction in CDCl_3_ in presence of 1 wt.% internal standard.

**Figure S16**. Time-resolved ^1^H NMR spectra recorded at different reaction times, showing the evolution of monomer and polymer resonances during polymerization of (a) poly(δ7) and (b) poly (δ0-δ7-δ0)

**Figure S17.** Monomer conversion as a function of reaction time during sequential homopolymerization and copolymerization.

**Figure S18**. ^1^H NMR Spectrum of (a) δ7 monomer, (b) poly(δ7) homopolymer, (c) δ0 monomer, and (d) poly(δ0-δ7-δ0) copolymer with detailed peak assignment. The unassigned peaks of poly(δ0-δ7-δ0) copolymer are for the poly(δ7) mid-block, and their detail can be found in part (b).

**Figure S19.** DSC heating traces of poly(δ7) and poly(δ0-δ7-δ0) copolymers with increasing molecular weight, showing a gradual shift in the glass transition temperature (Exo-up graph).

**Figure S20.** ^1^H NMR of Poly($\delta$7) and TMSPMA after (a) 2h, (b) 3h, (c) 4h, (d) 5h, and (e) 10h of reaction in CDCl3 in presence of 1 wt.% internal standard.

**Figure S21.** (a) Loss modulus and (b) Tan δ graphs for Poly(δ0-δ7-δ0) before and after dimethacrylation and in the presence of 20 wt.% IBOA.

**Table S1.** Young’s modulus and elongation at break of cured poly(δ7) and poly(δ0–δ7–δ0) networks with different MW formulated with 20 wt.% IBOA

| **Sample** | **Young’s Modulus**  **(MPa)** | **Elongation at Break**  **(%)** |
| --- | --- | --- |
| Poly($\delta$7) | 0.140 $\pm$ 0.012 | 237 $\pm$ 21 |
| Poly($\delta0-\delta$7$- \delta0$) – 9k | 1.83 $\pm$ 0.25 | 133 $\pm$ 18 |
| Poly($\delta0-\delta$7$- \delta0$) – 12k | 4.6 $\pm$ 0.16 | 168 $\pm$ 4 |
| Poly($\delta0-\delta$7$- \delta0$) – 17k | 38 $\pm$ 2.1 | 442 $\pm$35 |
| Poly($\delta0-\delta$7$- \delta0$) – 30k | 161 $\pm$ 11 | 234 $\pm$ 13 |

**Figure S22.** Tensile stress–strain curves of cured poly(δ7) and poly(δ0–δ7–δ0) networks with different molecular weights formulated with 20 wt.% BA.

**Figure S23.** Cyclic loading–unloading stress–strain responses of the DIW printed sample, illustrating repeatable mechanical behavior under successive deformation cycles.

**Figure S24.** Schematic and photograph of heated vat system designed to reduce the viscosity of resin for DLP 3D printing.

**Figure S25.** Cyclic loading–unloading stress–strain responses of the DLP printed sample, illustrating repeatable mechanical behavior under successive deformation cycles.

**Figure S26.** TGA and corresponding derivative weight loss DTG curve of the virging poly(δ0-δ7-δ0)-60k copolymer.

**Figure S27.** TGA and corresponding derivative weight loss DTG curve of the mechanically recycled poly(δ0-δ7-δ0)-60k copolymer.

**Figure S28.** Comparison of the ^1^H-NMR spectra with peak integral of (a) the residue remaining after distillation and depolymerization process of poly(δ0-δ7-δ0) and (b) the poly(δ0-δ7-δ0) copolymer right before depolymerization process.

**Figure S29.** Recycling efficiency of polymers formulated for DLP, DIW, and FFF.

**Figure S30.** Electrical resistance of conductive additives before and after recycling: (a) MWCNTs and (b) LiTFSI.

**Figure S31.** TGA and corresponding derivative weight loss DTG curve of the chemically recycled poly(δ0-δ7-δ0)-60k copolymer

**Figure S32**. DLP 3D printed lattice using Poly(δ0-δ7-δ0)-DMA + Poly(δ7)-2HEA (50:50). Both copolymer crosslinker and homopolymer macromonomer are synthesized using chemically recycled monomers. The polymerization and functionalization process for both polymers were same as the process for the original polymers synthesized using virgin monomer. Scale bar is 5mm.
